# Psychogenic polydipsia in dogs – a review of pathogenesis, diagnosis and treatment

**DOI:** 10.1111/jsap.70105

**Published:** 2026-02-13

**Authors:** G. Pavlovsky

**Affiliations:** ^1^ College of Veterinary Medicine, Dr. John A. Coyne South Clinic Veterinary Teaching Hospital, University of Illinois Urbana Illinois USA

## Abstract

Polyuria and polydipsia represent a common clinical presentation in dogs and may result from numerous disorders affecting different body systems. Compulsive water consumption is characteristic of psychogenic polydipsia, a primary polydipsia disorder rooted in neurologic, behavioural or environmental factors. While psychogenic polydipsia is relatively common and well described in people, little published information on its pathogenesis, diagnosis and treatment exists in dogs. Most dogs diagnosed with psychogenic polydipsia are young and systemically healthy, have significant polyuria and polydipsia, often have a nervous or anxious disposition, and may display stereotypical, compulsive or hyperactive behaviours. No specific test diagnoses psychogenic polydipsia definitively, and systematic elimination of other causes of polyuria and polydipsia is required. A multimodal approach to treatment may be most successful, including water restriction, environmental and behavioural modification, and pharmaceutical intervention.

## INTRODUCTION

Causes of polyuria and polydipsia (PU/PD) can be divided into primary polyuria with compensatory polydipsia or primary polydipsia with secondary polyuria. In dogs, psychogenic polydipsia (PPD) is a rare primary polydipsia disorder in which neurologic, behavioural or environmental changes result in compulsive water consumption leading to water diuresis and polyuria (Hall et al., 2021). Little published information exists regarding prevalence and pathogenesis of PPD in dogs. It is relatively common in humans, primarily affecting those with neurodevelopmental (*e.g*. autism, intellectual disabilities) and psychotic disorders (*e.g*. schizophrenia, schizoaffective disorder, bipolar disorder, obsessive‐compulsive disorder and psychotic depression) (Ahmadi & Goldman, 2020; Havens et al., 2022; Rangan et al., 2021).

In veterinary medicine, cases of primary polydipsia for which no other explanation has been found may be labelled as psychogenic, even in the absence of behavioural abnormalities (Henderson & Elwood, 2003; Olenick, 1999; Plickert et al., 2018; van Vonderen et al., 1999). As such, the terms psychogenic and primary polydipsia are often used interchangeably when diagnostic testing fails to confirm a specific cause. In human medicine, however, PPD is usually considered to be a distinct category of primary polydipsia disorders (Ahmadi & Goldman, 2020; Havens et al., 2022). Maintaining this distinction in veterinary medicine may be advisable, since confirmation of primary polydipsia in a dog with PU/PD does not necessarily equate with the PPD diagnosis. A diagnosis of primary polydipsia may be more appropriate in such cases until demonstrable signs of an underlying behavioural disorder could be linked to polydipsia.

According to the Merriam‐Webster dictionary, first use of the word polydipsia in the English language appeared in 1661 and was made in reference to insatiable thirst. Compulsive drinking of fluids was not described until the 19th century with the term dipsomania applied to those drinking excessive alcohol. Over the course of the 20th century, much research on PPD was published in human psychiatric literature, identifying a prevalence of 6% to 20% in both institutionalised psychiatric patients and those living in the community, frequently in association with a schizophrenia diagnosis (Deleon & Yorston, 2020; Navarro et al., 2017). A recent study evaluating clinical effects of hyponatraemia caused by excessive water intake associated with multiple aetiologies in 590 human adults reported PPD as the most common motivator (55% of all cases), mainly associated with the presence of schizophrenia spectrum disorder (Rangan et al., 2021). Another study reported the prevalence of PPD to be 15% to 25%, mostly in patients diagnosed with schizophrenia (Havens et al., 2022).

Literature review identified only eight studies (all are case reports) focusing on primary/psychogenic polydipsia in dogs and six in other animals (Table 1) (Bihannic & Desmarchelier, 2024; Buntain & Coffman, 1981; Fanton et al., 1987; Henderson & Elwood, 2003; Kim et al., 2025; Long et al., 2015; Marlois & Beata, 2017; Olenick, 1999; Pereira et al., 2025; Plickert et al., 2018; Pucci & Ottonetti, 2016; Rosenberg & Loomis, 1980; van Vonderen et al., 1999; Westerhof & Lumeij, 1988). Behaviour‐based aetiological investigation and management were described in only seven of these reports (Bihannic & Desmarchelier, 2024; Buntain & Coffman, 1981; Fanton et al., 1987; Marlois & Beata, 2017; Pereira et al., 2025; Pucci & Ottonetti, 2016; Westerhof & Lumeij, 1988).

**Table 1:** Veterinary reports on primary/psychogenic polydipsia

Primary author	Animal	Polydipsia diagnosis (reported cause)	Behaviour investigation
Bihannic, 2024	Dog	Psychogenic (post‐traumatic stress, contextual anxiety)	Yes
Buntain, 1981	Horse	Primary (salt intoxication)	Yes
Henderson, 2003	Dog	Primary (due to GI disease)	No
Fanton, 1987	Rhesus monkey	Psychogenic (triggered by abrupt caloric restriction)	Yes
Kim, 2015	Cat	Primary (unknown)	No
Long, 2015	Cat	Primary (unknown)	No
Marlois, 2017	Dog	Psychogenic (HSHA)	Yes
Olenick, 1999	Dog	Psychogenic (unknown)	No
Pereira, 2025	Dog	Psychogenic (anxiety, fear)	Yes
Plickert, 2018	Dog	Psychogenic (unknown)	No
Pucci, 2016	Dog	Psychogenic (chronic anxiety)	Yes
Rosenberg, 1980	Rhesus monkey	Psychogenic (stereotypic behaviour)	No
van Vonderen, 1999	Dog	Primary (unknown)	No
Westerhof, 1988	African grey parrot	Psychogenic (social deprivation)	Yes

This article describes comparative aspects of PPD, provides current theories on pathogenesis, outlines a practical diagnostic approach and therapeutic strategies. Clinical decision‐making reviewed here and steps ultimately leading to diagnosis of PPD may be helpful in evaluation of any dog presented with PU/PD.

## REGULATION OF WATER BALANCE

Arginine vasopressin (AVP), also known as antidiuretic hormone (ADH), is the main hormone responsible for maintenance of water balance via interaction with hypothalamus, pituitary gland and kidneys (Shiel, 2023a). AVP is produced in the hypothalamus, stored in the pituitary gland and is released in response to various stimuli (Shiel, 2023a). Half‐life of AVP is approximately 15 minutes, which enables rapid changes in blood concentrations and physiological effects (Shiel, 2023b). Successful interpretation of diagnostic testing for confirmed PU/PD requires an understanding of AVP effects on water consumption and urine production.

Movement of fluid between extracellular and intracellular spaces is determined by osmotic effect of solute, permeability of the membrane and hydrostatic pressure within the compartment. Certain small solutes (osmoles) present in high concentrations in physiologic fluids can affect water movement because they do not freely diffuse across biological membranes and thereby exert an osmotic effect across the membrane (DiBartola, 2012; Wellman et al., 2012). Osmolality is the number of osmoles per kilogram of solvent, whereas osmolarity is the number of osmoles per litre of solution (Wellman et al., 2012). In biological fluids (*e.g*. serum, urine), the difference between osmolality and osmolarity is negligible and osmolality is the term used most commonly. Sodium concentration is the principal determinant of serum osmolality (Wellman et al., 2012). A solution that is hyperosmotic has an osmolality greater than that of the reference solution (*e.g*. plasma) (DiBartola, 2012). Hypoosmotic solutions have lower osmolality, and isosmotic solutions have the same osmolality as the reference solution (DiBartola, 2012). In serum, solutes in greatest concentration include glucose, urea, bicarbonate, sodium, potassium and chloride (Wellman et al., 2012). Only glucose and sodium are effective osmoles because the others freely diffuse across most cell membranes (Wellman et al., 2012). Large molecules (*e.g*. albumin) have a negligible effect on fluid movement and account for only 0.5% of total osmotic pressure (Wellman et al., 2012). Measurement of osmolality via freezing‐point depression osmometry is more accurate than vapour point determination (DiBartola, 2012; Wellman et al., 2012).

Volume of extracellular fluid (ECF) is maintained by total body sodium content, while osmolality and sodium concentration are affected by water balance (DiBartola, 2012). Kidneys play a central role because they normally excrete the same amount of sodium as ingested each day (DiBartola, 2012; Shiel, 2023b). Glomerular filtration and tubular reabsorption are the mechanisms involved in sodium balance (DiBartola, 2012; Shiel, 2023b).

**FIG. 1:**
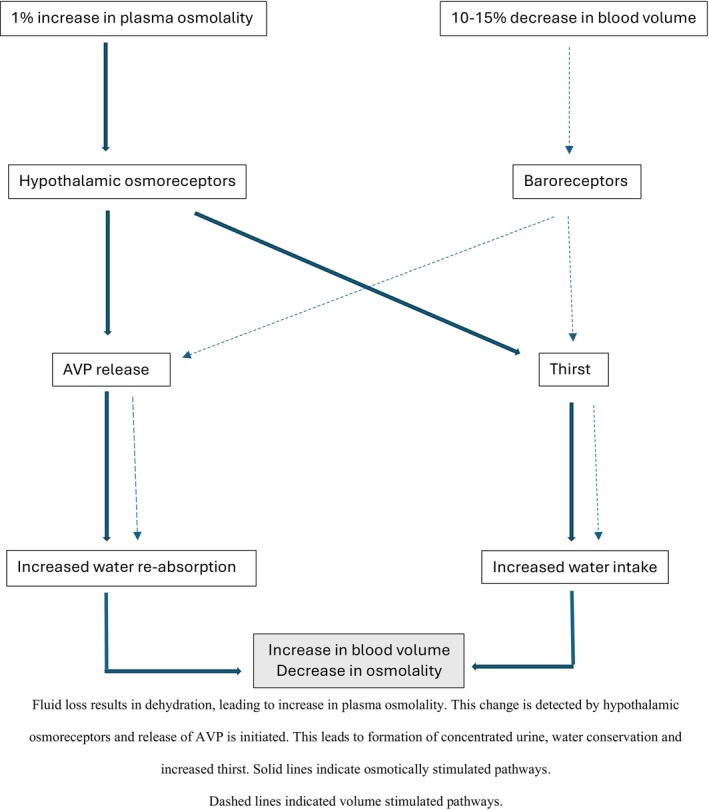
Primary mechanisms involved in regulation of water balance.

Dehydration can be classified by type of fluid lost from the body (DiBartola, 2012). Free water or hypotonic fluid loss results in hypertonic dehydration and is common with inadequate fluid intake (DiBartola, 2012). When the resultant increase in plasma osmolality is detected by hypothalamic osmoreceptors, release of AVP is initiated, resulting in formation of concentrated urine, water conservation and sensation of thirst (Fig. 1) (Shiel, 2023a, 2023b). As little as a 1% increase in plasma osmolality triggers AVP release and water resorption in renal tubules, increasing urine concentration and decreasing urine volume (Shiel, 2023b).

Other factors that may alter secretion of and/or sensitivity to AVP include blood pressure changes detected by baroreceptors in the carotid sinus and aortic arch; reduction in blood volume sensed by receptors in pulmonary veins and atria; certain drugs; the act of drinking; various metabolic and endocrine disorders; and nonspecific factors such as nausea, pain, exercise and emotion (Shiel, 2023b). Compared to the minimal rise in plasma osmolality, at least 10% to 15% blood volume or pressure reduction is needed to stimulate AVP release (Shiel, 2023b).

AVP binds to the basolateral membrane of principal renal tubular cells, activating aquaporin‐2 water channels, which causes an increase in water permeability, movement of free water from the tubular lumen into the hypertonic renal medulla and concentrated urine (Nielsen et al., 1995; Shiel, 2023b; van Vonderen, Kooistra, et al., 2004). For concentrated urine to form, three factors must be in place: (1) adequate secretion of AVP and renal tubular cell sensitivity and response; (2) the presence of at least one‐third of functional renal mass; and (3) maintenance of medullary hypertonicity (Shiel, 2023a).

Diuresis is urine flow that is greater than normal, typically defined as exceeding 1 to 2 mL/kg/hour in dogs, and can be defined in terms of the underlying cause (DiBartola, 2012). Water diuresis is increased urine flow caused by decreased reabsorption of free water in renal collecting ducts that results in hypoosmotic urine. It is a feature of diabetes insipidus and primary polydipsia (DiBartola, 2012). Solute or osmotic diuresis is increased urine flow caused by decreased reabsorption of solutes within renal tubules (*e.g*. glucose). It can occur with diabetes mellitus and administration of mannitol and other osmotically active drugs. With osmotic diuresis, urine osmolality approaches that of plasma (DiBartola, 2012).

## THEORIES ON PATHOGENESIS

Exact mechanisms responsible for polydipsia in dogs with PPD are unknown. Under normal physiological conditions, excessive thirst and an increase in water consumption inhibit AVP production and the resultant decrease in osmolality also suppresses AVP secretion (Shiel, 2023b; van Vonderen et al., 1999). Central diabetes insipidus (CDI) develops when the circulating concentration of AVP is deficient; nephrogenic diabetes insipidus (NDI) is characterised by inadequate response to normal AVP concentration (DiBartola, 2012; Shiel, 2023a, 2023b). Data on AVP secretion in dogs with primary polydipsia are limited and contradictory, although abnormal AVP release has been documented in several studies (Biewenga et al., 1987; Mulnix et al., 1976; van Vonderen et al., 1999; van Vonderen, Kooistra, et al., 2004; van Vonderen, Wolfswinkel, et al., 2004).

In humans, proposed mechanisms of primary polydipsia include: (1) reset of AVP osmotic set point in the hypothalamus to a lower threshold caused by psychosis and acute psychological stress; (2) genetic abnormalities involving polymorphisms in the dopaminergic D2 receptor (D2R2) gene; (3) dopamine‐influenced motivated behaviours and learning; and (4) abnormalities in renin–angiotensin–aldosterone (RAAS) and sympathetic nervous systems (SNS) (Ahmadi & Goldman, 2020; Cabib et al., 1997; Havens et al., 2022). Animal models suggest involvement of dopaminergic D2 receptors in propagation of polydipsia (Ahmadi & Goldman, 2020; Moreno & Flores, 2012). It is unclear which, if any, of these mechanisms is responsible for primary polydipsia in dogs (Henderson & Elwood, 2003; Shiel, 2023a). When comparative data on PPD are considered, however, psychological stress and behavioural abnormalities emerge as common factors in both humans and animals affected with the condition (Ahmadi & Goldman, 2020; Bihannic & Desmarchelier, 2024; Buntain & Coffman, 1981; Fanton et al., 1987; Hall et al., 2021; Havens et al., 2022; Marlois & Beata, 2017; Moreno & Flores, 2012; Navarro et al., 2017; Pereira et al., 2025; Pucci & Ottonetti, 2016; Shiel, 2023a; Westerhof & Lumeij, 1988). Thus, the presence of a documented behavioural disorder constitutes an essential element in diagnosing PPD in dogs.

Major categories of primary polydipsia‐related disorders in humans include: (1) psychosis‐intermittent hyponatraemia‐polydipsia syndrome; (2) autism, intellectual disability, bipolar and psychotic depression disorders; (3) compulsive water drinking/PPD; (4) dipsogenic diabetes insipidus (DI); (5) brain injury or disease, especially of the hypothalamus; and (6) health enthusiasts and athletes who believe consumption of water in excess of need promotes cognition and performance (Ahmadi & Goldman, 2020). Very few affected patients have disruptions in homeostatic regulation of water balance (*i.e*. hypovolaemia, hypernatraemia, hypotension), and their thirst intensity is likely the product of psychological or cognitive influences (Ahmadi & Goldman, 2020). Distinct physiological alterations in water balance have been demonstrated in these disorders and reflect the impact of various cognitive and emotional (*i.e*. non‐homeostatic) factors on water intake and satiation (Ahmadi & Goldman, 2020).

Psychogenic polydipsia has been documented in nervous or anxious dogs with concurrent compulsive and hyperactivity disorders (Hall et al., 2021; Marlois & Beata, 2017; Pereira et al., 2025). These dogs may become clinical following stress‐inducing events, similar to the reported cases in human psychiatric literature (Hall et al., 2021; Marlois & Beata, 2017). Compulsive behaviours are defined as excessive, repetitive and goal‐oriented activities (Denenberg, 2021a). Once the goal is achieved (*e.g*. water consumption), dopamine, beta‐endorphins and endogenous opioids are released, providing strong reinforcement (Denenberg, 2021a).

Hyperactive behaviour (*i.e*. hypersensitivity–hyperactivity syndrome/HSHA) in dogs has been linked with human attention deficit and hyperactivity disorder (ADHD) (Bleuer‐Elsner et al., 2021; Denenberg, 2021a; Marlois & Beata, 2017). Dogs suffering from severe HSHA typically display three main abnormal behaviours: hyper‐reactivity, impulsivity and attention deficit (Bleuer‐Elsner et al., 2021; Denenberg, 2021a). While ADHD in humans has not been associated with abnormalities in water consumption, one report of a dog with HSHA suggested it as a cause of PPD and the dog improved with behavioural therapy and anxiolytic medication (Marlois & Beata, 2017).

When considering management of underlying behavioural disorders as part of therapy, the question is whether the stress‐inducing event alone (social or situational factor) is enough to induce the behaviour or whether some predisposition (innate, biological factor) must be present (Ahmadi & Goldman, 2020; Cabib et al., 1997). A concept in psychiatry and psychopathology, termed the diathesis‐stress model, explains how both factors interact with each other to give rise to a disorder. According to this model, development of a psychological condition requires pre‐existence of a diathesis, that is, an innate predisposition to the condition (Ahmadi & Goldman, 2020; Cabib et al., 1997). Stress, a set of challenging circumstances, may then trigger the condition (Ahmadi & Goldman, 2020; Cabib et al., 1997). Animal models support the theory of stress diathesis producing distinct mental disorders in polydipsic schizophrenic humans with increased drinking and other compulsive or stereotypical behaviours (Ahmadi & Goldman, 2020). These behaviours do not serve homeostatic needs but function as “primitive” coping mechanisms that simultaneously impair more complex coping responses (Ahmadi & Goldman, 2020). Studies in rats support this influence of stress on expression of behaviour and have been used as a model for compulsive neuropsychiatric disorders associated with PPD in humans (Cabib et al., 1997; Navarro et al., 2017).

Marked increase in water intake associated with primary polydipsia in dogs can result from altered response of brain thirst centres to appropriate neural, hormonal or osmoregulatory stimuli that are not normal compensatory mechanisms for primary polyuria (Shiel, 2023b). Polydipsia in dogs is defined as fluid intake greater than 90 to 100 mL/kg/day (Shiel, 2023a). Production of urine greater than 50 mL/kg/day defines polyuria (Mulnix et al., 1976; Shiel, 2023a). Polyuria is defined as production of urine greater than 50 mL/kg/day and reportedly up to 722 mL/kg/day (Mulnix et al., 1976; Shiel, 2023a). Primary polydipsia may produce no clinical signs other than a noticeable increase in thirst and urination. In severe cases, however, it may lead to significant hyponatraemia and life‐threatening complications (Plickert et al., 2018; Rangan et al., 2021). Prevalence of clinical hyponatraemia secondary to PPD in dogs is unknown but hyponatraemia occurs in 3% to 6% of humans diagnosed with chronic schizophrenia (Ahmadi & Goldman, 2020; Rangan et al., 2021).

## DIAGNOSTIC PROCESS

Initial diagnostic testing is aimed at ruling out common causes of PU/PD and differentiating primary polydipsia from primary polyuria. Whether primary polydipsia is then classified as psychogenic depends on the presence of associated behavioural disorder(s), rather than by simply ruling out other conditions causing primary polyuria. Differential diagnoses for PU/PD are summarised in Table 2 (DiBartola, 2012; Shiel, 2023a). Detailed questioning helps direct the diagnostic process. Certain diseases associated with PU/PD are more prevalent in specific age groups and breeds and may manifest with concurrent clinical signs or laboratory findings indicative of those diseases. Note that in some dogs, confirmation of primary polydipsia may be complicated by the presence of abnormalities contributing to clinical signs, such as urethral sphincter mechanism incompetence. Cases of primary polydipsia concurrent with less common conditions (ectopic ureter, portosystemic shunt) have also been described (Bihannic & Desmarchelier, 2024; Pereira et al., 2025).

**Table 2:** Differential diagnoses for polyuria and polydipsia in dogs

	Condition
Primary PD	Dipsogenic diabetes insipidus Gastrointestinal disease Hepatic failure Psychogenic polydipsia
Primary PU	Acromegaly Bacterial endotoxins (*e.g*. pyometra) Anticonvulsants (*e.g*. phenobarbital) Chronic kidney disease^‡^ Congenital or familial primary nephrogenic diabetes insipidus (NDI) Cranial trauma Diabetes mellitus^‡^ Diuretics (*e.g*. mannitol, furosemide) Ethylene glycol toxicosis Glucocorticoids (*e.g*. prednisone) Hepatic disease (*e.g*. portosystemic shunts) Hypercalcemia Hypercortisolism^‡^ Hyperthyroidism Hypoadrenocorticism Hypokalaemia Idiopathic (congenital) central diabetes insipidus (CDI) Intracranial neoplasia Leptospirosis, atypical Polycythaemia Post‐hypophysectomy Post‐obstructive diuresis Primary hyperaldosteronism Primary renal glucosuria (*e.g*. Fanconi syndrome) Pyelonephritis Salt supplementation

^‡^ Most common.

Presenting complaint typical of PPD is PU/PD persisting for months to years with no other signs of illness (Denenberg, 2021a; Pereira et al., 2025; Plickert et al., 2018). No age or gender predisposition has been noted. A history of nervous or anxious temperament and onset of polydipsia during or following stressful events are commonly reported (Bihannic & Desmarchelier, 2024; Marlois & Beata, 2017; Pereira et al., 2025; Pucci & Ottonetti, 2016). History of recent trauma, environmental changes or chronic behavioural problems, such as aggressiveness, compulsive repetitive activities, anxiety, noise phobias or fearfulness during hospital visits may be present (Bihannic & Desmarchelier, 2024; Denenberg, 2021a; Hall et al., 2021; Pereira et al., 2025; Plickert et al., 2018; Pucci & Ottonetti, 2016). A detailed behavioural history can uncover behaviours that some dog owners consider acceptable, minimally disruptive and tolerable, opting not to mention them unless prompted. However, certain such behaviours may be indicative of chronic anxiety, fear, reactivity, poor impulse control, obsessive‐compulsive and other behavioural disorders tied to PPD, increasing the index of suspicion for the condition. Urinary incontinence and inappropriate urination may occur because of the large volume of urine produced.

Physical examination findings are unremarkable in most dogs with PPD. As long as water is not restricted, hydration status and subjective measures of peripheral perfusion (*e.g*. mucous membrane colour, capillary refill time) are normal. Abnormalities such as altered hydration status, muscle mass or body condition, organomegaly, lymphadenopathy or signs of abdominal pain may raise suspicion of a different disease process. Affected dogs may display signs of fear, anxiety, stress and displacement behaviours, for example, pacing, lip licking, yawning, vigilance, pinning of pinnae, lowering of head and hiding.

While no specific laboratory abnormalities are diagnostic of primary polydipsia, demonstration of low urine specific gravity (USG) and ability to concentrate urine following water restriction are highly suggestive, once other causes of PU/PD have been ruled out (Shiel, 2023b; van Vonderen et al., 1999). It is valuable to develop a stepwise process to rule out most common causes of PU/PD for several reasons. First, numerous diseases and conditions can result in PU/PD, with several being much more common than PPD. Second, some conditions in their classic forms are often associated with specific abnormalities, which may be easily identified with simple testing, precluding more expensive, invasive or complicated diagnostic work‐up. Finally, reports of PU/PD may be somewhat subjective, and it is important to confirm that the presenting problem actually exists (Shiel, 2023a). This may be done by collecting a complete history regarding water intake and urination and measuring USG on several random samples to document inadequate urine concentration, for example, hyposthenuria or isosthenuria (Shiel, 2023a).

In situations when PU/PD is reported but USG is consistently normal (>1.030), owners can be instructed to measure water intake at home over a period of several days (Shiel, 2023a). While PU/PD is highly likely if average intake is documented to be greater than 100 mL/kg/day, polydipsia may manifest itself at much lower daily volumes in dogs who normally drink little. Among healthy dogs, water consumption may vary considerably and is dependent on activity levels, type of diet (moist vs. dry), environmental conditions and other factors (O'Connor & Potts, 1969; Ramsay & Thrasher, 1991). For example, a wide range of water consumption, from 4 to 109 mL/kg/day, has been reported in normal laboratory dogs (O'Connor & Potts, 1969). Additionally, one study found the upper limit of normal daily water consumption to be approximately 70 mL/kg/day, quite a bit less than the commonly used 100 mL/kg/day figure (Ramsay & Thrasher, 1991). Thus, using a specific water intake cut‐off in determining whether polydipsia is present could be misleading in those dogs who do not reach 100 mL/kg/day. In many cases, history will provide adequate information to confirm that PU/PD is present: many dogs will have a noticeable increase in water consumption compared to their baseline and will either urinate indoors or alert their owners to be taken outside more frequently, producing a larger quantity of urine.

Urine osmolality provides the most accurate assessment of urine concentration but is difficult to perform in practice (Ayoub et al., 2013; Rudinsky et al., 2019; Watson, 1998). USG is a measure of relative density of urine in relation to deionised water as measured by a refractometer, an instrument that refracts light based on density of dissolved solids in urine (DiBartola, 2012; Rudinsky et al., 2019; Watson, 1998). Changes in USG generally correlate well with changes in urine osmolality, that is, the higher the USG, the higher urine osmolality and vice versa (Ayoub et al., 2013). This relationship is true as long as no large molecules are present in urine (*e.g*. glucose) that may change the refractive index (Ayoub et al., 2013; DiBartola, 2012; Watson, 1998). A study of 100 dogs compared USG read by four different refractometers with urine osmolality measured with freezing‐point depression finding significant correlation between each refractometer and urine osmolality measurements, but some variability in USG values between the different devices (Rudinsky et al., 2019). A wide USG range is possible in healthy, well‐hydrated dogs and different reference intervals for adequate concentrating ability and isosthenuria are reported in the literature. Generally, a USG >1.030 indicates adequate urine concentration (Shiel, 2023b; Watson, 1998). In dogs with free access to water, finding of USG >1.030 makes polydipsia unlikely, however, when water restricted, dogs with primary polydipsia can achieve a USG of 1.030 or higher, in contrast to other causes of PU/PD.

Inadequately concentrated urine (USG <1.030) may be within isosthenuric range (1.008 to 1.012) or be hyposthenuric (<1.008) (Shiel, 2023b; Watson, 1998). Isosthenuria is characteristic of renal disease in azotaemic dogs because it indicates inability of the kidneys to concentrate or dilute the urine. In most renal diseases, however, the loss of functional nephron mass is gradual so any USG value in isosthenuric and inadequate ranges in azotaemic or dehydrated dogs is suggestive of primary renal failure (Shiel, 2023b; Watson, 1998). Hyposthenuria indicates that kidneys can dilute the urine but are unable to concentrate it, that is, loop of Henle and proximal renal tubules are functional, but connecting tubules are not (Shiel, 2023b; Watson, 1998). This happens either because of AVP deficiency or decreased responsiveness to or inhibition of AVP (Shiel, 2023b; Watson, 1998).

Once PU/PD is established, fasting biochemistry panel, complete blood count and complete urinalysis (UA) are indicated. Urine culture may be considered a part of the initial database, especially if clinical or laboratory signs of urinary tract inflammation are present. Haematological and biochemistry parameters are typically normal in dogs with primary polydipsia, unless dehydration is present. Mild hyponatraemia and hypokalaemia may occur in up to 20% of dogs (Ueda et al., 2015). The most notable and often only abnormality is low USG (Shiel, 2023a; van Vonderen et al., 1999; van Vonderen, Wolfswinkel, et al., 2004; Watson, 1998). Urine is usually extremely dilute (*i.e*. USG <1.008); however, USG can fluctuate (Watson, 1998).

Depending on physical examination findings and abnormalities discovered in initial tests, other testing may include cortisol assays for suspected hypercortisolism or hypoadrenocorticism; thyroid assays to rule out hyperthyroidism; imaging studies of chest and/or abdomen to identify potential causes of hypercalcaemia, altered renal parameters, palpable abdominal masses, lymphadenopathy or evidence of other diseases (*e.g*. pyometra, ectopic ureter, portosystemic shunt); serological tests for infectious diseases (*e.g*. leptospirosis); and bile acids for possible hepatic insufficiency (Bihannic & Desmarchelier, 2024; Pereira et al., 2025; Shiel, 2023a). Measurement of acute phase proteins (*e.g*. C‐reactive protein [CRP]), progesterone and anti‐Müllerian hormone may be indicated if stump pyometra is suspected (Janković et al., 2022).

Once disorders associated with primary polyuria (*i.e*. diseases causing secondary NDI and osmotic diuresis) have been ruled out, presumptive diagnosis of primary polydipsia can be made by differentiating it from CDI and primary NDI (Table 2) (Shiel, 2023a, 2023b; van Vonderen et al., 1999; van Vonderen, Wolfswinkel, et al., 2004). Differentiation can be accomplished by demonstrating adequate urine concentrating ability in response to desmopressin (DDAVP) administration or water deprivation test (WDT) and measuring plasma and urine osmolality (DiBartola, 2012; Hall et al., 2021; Mulnix et al., 1976; Nichols & Hohenhaus, 1994; Pereira et al., 2025; Plickert et al., 2018; Shiel, 2023a, 2023b).

Since AVP production varies with different aetiologies of PU/PD, measuring plasma AVP levels may seem beneficial (Biewenga et al., 1987; van Vonderen, Wolfswinkel, et al., 2004). Theoretically, AVP concentrations are expected to be undetectable with complete CDI; normal with NDI and primary polydipsia; and low with partial CDI (Biewenga et al., 1987; van Vonderen, Wolfswinkel, et al., 2004). However, very few studies correlating AVP production with specific aetiology have been performed in dogs. Additionally, secretion of AVP is variable and the availability of commercial AVP assays is limited (Biewenga et al., 1987; Mulnix et al., 1976; Scollan et al., 2013; van Vonderen et al., 1999; van Vonderen, Wolfswinkel, et al., 2004).

Clinical use of WDT is limited to hyposthenuric patients in whom CDI, primary NDI or primary polydipsia are strongly suspected and common causes of PU/PD have been ruled out (Hall et al., 2021; Mulnix et al., 1976; Plickert et al., 2018; Shiel, 2023a). Contraindications to WDT include azotaemic or severely polyureic dogs (DiBartola, 2012; Plickert et al., 2018; Shiel, 2023a). WDT is not helpful in dehydrated dogs with dilute urine because this combination of findings constitutes a failed test.

WDT is labour‐intensive, difficult to perform correctly, stressful, associated with potential complications and difficult to interpret (dogs with hypercortisolism may have a positive response) (Mulnix et al., 1976; Plickert et al., 2018; Shiel, 2023a).

Before WDT is considered in a dog with suspected CDI, NDI or primary polydipsia, DDAVP administration trial is typically used as a safer and less invasive alternative (Nichols & Hohenhaus, 1994; Pereira et al., 2025; Plickert et al., 2018; Shiel, 2023b). DDAVP administration is safe to perform and can be conducted at home with owners carefully monitoring water intake and watching for complications, for example, water intoxication (Nichols & Hohenhaus, 1994; Shiel, 2023b). USG is measured on day 1, then DDAVP is administered in tablet form at 0.1 to 0.2 mg/dog PO q 8 h for 5 to 7 days and USG is measured on at least two random samples between days 5 and 7 (Pereira et al., 2025; Rayalam et al., 2009). Alternatively, an intranasal DDAVP preparation can be administered (1 to 4 drops) into the conjunctival sac q 12 h for 5 to 7 days (Nichols & Hohenhaus, 1994; Rayalam et al., 2009). A greater than 50% USG increase over baseline by 7 days, especially if greater than 1.030 and noticeable reduction in PU/PD are consistent with CDI (Shiel, 2023b).

Dogs with NDI typically show no response to DDAVP, and dogs with primary polydipsia have only a slight decrease in water consumption (Pereira et al., 2025; Shiel, 2023b). However, because some dogs with primary polydipsia have chronically low serum osmolality that may have suppressed AVP production, mild improvement in urine concentration may occur with DDAVP (Shiel, 2023b). Persistent polydipsia in dogs who fail to respond to DDAVP (primary polydipsia) may lead to water intoxication (hypo‐osmolar hyponatraemia) that manifests as weakness, lethargy, nausea, tremors, seizures and coma (Nichols & Hohenhaus, 1994; Plickert et al., 2018; Shiel, 2023b). These side effects resolve with discontinuation of DDAVP (Nichols & Hohenhaus, 1994; Shiel, 2023b).

When WDT is deemed necessary, gradual WDT may be preferable to the traditionally used modified WDT, because it can be performed at home, does not involve complete water restriction and is less labour intensive (Foster, 2023). Gradual WDT differs from the modified WDT in the following ways: (1) phase 1 involves measurement of 24‐hour water consumption at home, repeated over 3 to 5 days to obtain average intake and (2) water restriction is implemented slowly at home, at a rate of 5% per day with patient examined and monitored in hospital daily (Foster, 2023). Laboratory assessment, monitoring, test endpoints and interpretation are implemented as in phases 2 and 3 of modified WDT (Table 3) (Shiel, 2023b).

**Table 3:** Modified water deprivation test and expected outcomes

**Phase 1: Gradual water intake restriction**
Amount of water given is restricted over a 3‐ to 5‐day period until 60 to 80 mL/kg/day Owners monitor for any signs of illness USG is measured at conclusion of this phase (USG > 1.030 supportive of primary polydipsia and WDT no longer needed)
**Phase 2: Water deprivation**
Patient is admitted to the hospital, weighed and assessed physically Indwelling urinary catheter is placed; no food or water is given Blood urea nitrogen (BUN), creatinine, electrolytes, packed cell volume (PCV) and total solids (TS) measured Bladder is emptied, USG is checked, and weight obtained every hour BUN, creatinine, electrolytes, PCV and TS checked every 4 to 6 hours Test stopped if: 5% loss of body weight Evidence of clinical dehydration, vomiting, lethargy or altered mentation Azotaemia USG >1.030
**Phase 3: Administration of DDAVP (not needed if administered as a test before WDT)**
If patient is 5% dehydrated and urine is not adequately concentrated, DDAVP is administered Mentation, weight, BUN, creatinine, electrolytes, PCV and TS assessed Bladder is emptied DDAVP is administered at 2 to 10 mcg IV or an intranasal DDAVP formulation is administered into conjunctival sac (1 to 4 drops per dog) Bladder is emptied in 30 minutes and USG is measured; repeat every 30 minutes for up to 2 hours until USG >1.015; if USG remains <1.015, repeat every hour for up to 8 hours until USG >1.015 Water is re‐introduced slowly (10 to 20 mL/kg every 30 minutes, for 2 hours)
**Expected results**
USG >1.030 after Phase 1 indicates primary (psychogenic) polydipsia USG <1.008 after Phase 1 indicates either complete CDI or primary NDI USG >1.015 after DDAVP administration indicates partial CDI

Dogs with CDI and NDI do not concentrate their urine with water restriction, while dogs with primary polydipsia can attain adequate urine concentration. If DDAVP trial has not yet been performed, WDT can be followed by administration of DDAVP when USG remains low despite water deprivation. Dogs with CDI respond to DDAVP; dogs with NDI do not (Nichols & Hohenhaus, 1994; Shiel, 2023b). Dogs with primary polydipsia do not respond or respond only minimally (Nichols & Hohenhaus, 1994; Plickert et al., 2018; Shiel, 2023b).

Patients with partial CDI and those with primary polydipsia are difficult to differentiate. Dogs with both conditions may initially achieve similar levels of urine concentration following water deprivation, and USG does not substantially improve with administration of DDAVP as occurs with complete CDI (Nichols & Hohenhaus, 1994). Dogs with primary polydipsia have adequate circulating levels of AVP, so their response to DDAVP is minimal. Dogs with partial CDI may not be as responsive to an exogenous source (*e.g*. DDAVP) as dogs with complete CDI, theoretically because they have been sensitised to low levels of circulating AVP (Nichols & Hohenhaus, 1994; Shiel, 2023b). No single measure of urine concentration (*i.e*. USG or urine osmolality) or percentage increase following DDAVP administration can definitively distinguish between the partial CDI and PPD (Nichols & Hohenhaus, 1994; Shiel, 2023b).

Random paired measurement of urine and plasma or serum osmolality can provide supportive evidence of primary polydipsia in dogs with unrestricted access to water (van Vonderen et al., 1999; van Vonderen, Kooistra, et al., 2004). Plasma osmolality in healthy dogs has a wide range, reported as 280 to 320 pOsm (DiBartola, 2012; van Vonderen et al., 1999; van Vonderen, Kooistra, et al., 2004). Dogs with CDI and NDI have primary polyuria with compensatory polydipsia in which renal loss of free water leads to reduction of blood volume and increased plasma osmolality. Dogs with CDI are expected to have high normal or elevated plasma osmolality and decreased urine osmolality, especially if water intake is lower than urinary output (van Vonderen et al., 1999; van Vonderen, Kooistra, et al., 2004). Long‐term increased water intake associated with primary polydipsia leads to increased blood volume, decreased plasma osmolality and decreased AVP secretion with production of large volumes of urine (van Vonderen et al., 1999; Watson, 1998). Dogs with persistent primary polydipsia should have low normal or decreased plasma osmolality and low urine osmolality (Shiel, 2023b; van Vonderen et al., 1999; van Vonderen, Kooistra, et al., 2004). If a dog has free access to water and has random plasma osmolality <280 pOsm, primary polydipsia is the more likely cause than CDI or NDI (Shiel, 2023b).

In summary, low USG that may fluctuate, failure to respond to DDAVP administration, low or low‐normal plasma osmolality with low urine osmolality and a relatively long time for dehydration to occur with water restriction support the diagnosis of primary polydipsia (DiBartola, 2012; Shiel, 2023a, 2023b). Concurrent presence or a history of behavioural abnormalities make primary polydipsia more likely to be psychogenic in origin (Hall et al., 2021; Pereira et al., 2025). It is worth noting that, PU/PD may be intermittent (*e.g*. during stressful episodes), so laboratory findings may not consistently fit the results expected for primary polydipsia.

## TREATMENT OF PSYCHOGENIC POLYDIPSIA

Human compulsive water drinkers (primary polydipsia group) mostly closely resemble dogs with PPD (Ahmadi & Goldman, 2020; Hall et al., 2021). These human patients frequently respond to treatment of an underlying psychiatric disorder, but the efficacy of pharmacotherapeutic management is questionable (Ahmadi & Goldman, 2020; Hall et al., 2021). Humans with ADHD, a model for canine HSHA, may respond to selective serotonin reuptake inhibitors (SSRIs), and two reports support the use of high dose fluoxetine for HSHA in dogs (Bleuer‐Elsner et al., 2021; Carlisi et al., 2016; Masson & Gaultier, 2018). Randomised, controlled trials of treatment of PPD in dogs are lacking. Because of associated behavioural problems, a thorough history and a behavioural diagnosis are essential in developing an effective treatment plan. Treatment of PPD involves (1) water restriction, (2) environmental modification, (3) behavioural modification and (4) pharmaceutical intervention (Bihannic & Desmarchelier, 2024; Pereira et al., 2025).

Gradual limitation of water intake, that is, 10% reduction weekly until an amount equal to 60 to 80 mL/kg per day is reached, improves and may resolve PU/PD (Bihannic & Desmarchelier, 2024). One report of a dog with PPD described significant improvement in both behavioural condition and water intake without intentional restriction of water access (Pereira et al., 2025).

Behavioural and environmental modification are started simultaneously and tailored to the underlying psychological disorder. Since PPD in dogs may be a compulsive disorder, treatment aims to manage perpetuating environmental, psychological and physical factors associated with the underlying compulsion (Bihannic & Desmarchelier, 2024; Denenberg, 2021a; Pereira et al., 2025). Exact approach varies based on specifics of the individual patient and generally includes (1) removing triggers of behaviours associated with anxiety and arousal; (2) reinforcement of calm behaviour; (3) response substitution; (4) maintaining positive and predictable interactions; and (5) optimisation of the environment and daily routine to meet patient needs (Bihannic & Desmarchelier, 2024; Denenberg, 2021a; Hall et al., 2021; Marlois & Beata, 2017; Pereira et al., 2025).

In conjunction with a behavioural/environmental modification plan, pharmaceutical intervention is often necessary for managing compulsive and HSHA disorders (Bihannic & Desmarchelier, 2024; Bleuer‐Elsner et al., 2021; Denenberg, 2021b; Masson & Gaultier, 2018). Serotonergic medications are considered first‐line therapy (Bihannic & Desmarchelier, 2024; Bleuer‐Elsner et al., 2021; Denenberg, 2021b; Masson & Gaultier, 2018; Pereira et al., 2025). Serotonergic agents include SSRIs (*e.g*. fluoxetine, sertraline, paroxetine); tricyclic anti‐depressants (*e.g*. amitriptyline, clomipramine); serotonin–norepinephrine reuptake inhibitors (*e.g*. duloxetine); selective antagonist and reuptake inhibitors (*e.g*. trazodone); and alpha‐2 antagonists (*e.g*. mirtazapine) (Denenberg, 2021b). SSRIs, namely fluoxetine, are the most commonly used serotonergic agents in animals (Denenberg, 2021b). Given the role serotonin plays and its effect on the brain, SSRIs exert a broad range of effects that benefit a variety of conditions, including anxiety disorders, fears, compulsive disorders, hyperactivity, impulsivity and depression. All these conditions may be associated to some degree with PPD (Denenberg, 2021b; Hall et al., 2021; Plickert et al., 2018). Fluoxetine is given at 1 to 2 mg/kg po q 24 h for fearfulness, anxiety, aggression, depression, phobias, compulsive behaviours, high arousal and impulsivity. Higher doses (2 to 4 mg/kg po q 24 h) have been used for HSHA (Bleuer‐Elsner et al., 2021; Denenberg, 2021b; Marlois & Beata, 2017). Use of fluoxetine specifically for treatment of PPD has been reported with dosing ranging from 1.5 mg/kg to 2.3 mg/kg PO q 24 h (Bihannic & Desmarchelier, 2024; Pereira et al., 2025). After initiation of a comprehensive treatment plan, water restriction may no longer be necessary, and medication may also be discontinued in some cases (Bihannic & Desmarchelier, 2024).

## PROGNOSIS

Prognosis has been reported as excellent but seeing improvement may take a long time (Bihannic & Desmarchelier, 2024; Pereira et al., 2025). Water restriction alone may be effective in resolving PU/PD and management of underlying behavioural problems helps reduce incidence of relapse. Client education focuses on provision of a specific amount of water, management of expectations and treatment of any underlying behavioural disorder (Denenberg, 2021a; Pereira et al., 2025). Behavioural management must be comprehensive, with frequent client communications and adjustment of therapy (Denenberg, 2021a). Monitoring consists of owner assessments of water consumption and urination frequency as well as improvement of underlying behavioural disorder.

### Author contributions

**G. Pavlovsky:** Conceptualization; investigation; writing – original draft; writing – review and editing; resources; supervision; formal analysis.

### Conflict of interest

No conflicts of interest have been declared.

## Data Availability

Data sharing is not applicable to this article as no datasets were generated or analysed during the current study.
